# Multiple targets related to mitochondrial function unveiled by metabolomics and proteomics profiles of hearts from atrial fibrillation patients

**DOI:** 10.3389/fphys.2023.1123391

**Published:** 2023-04-04

**Authors:** Weizhuo Liu, Bo Hu, Yuliang Wang, Xiaobin Zhang, Miao Zhu, Yu Shi, Changfa Guo, Yangyang Zhang

**Affiliations:** ^1^ Department of Critical Care Medicine, Shanghai Chest Hospital, Shanghai Jiao Tong University School of Medicine, Shanghai, China; ^2^ Department of Cardiology, Shanghai East Hospital, School of Medicine, Tongji University, Shanghai, China; ^3^ Department of Immunology, Nanjing Medical University, Nanjing, China; ^4^ Department of Cardiovascular Surgery, Shanghai Chest Hospital, Shanghai Jiao Tong University School of Medicine, Shanghai, China; ^5^ Department of Cardiovascular Surgery, Zhongshan Hospital, Fudan University, Shanghai, China; ^6^ Department of Cardiovascular Surgery, Shanghai East Hospital, Tongji University School of Medicine, Shanghai, China

**Keywords:** atrial fibrillation, proteomics, metabolomics, mitochondrial function, multiple targets

## Abstract

**Background:** The prominent mitochondrial metabolic changes of the atrium reportedly have significant impact on electrical signals and structural remodeling which play important roles in the occurrence and development of atrial fibrillation (AF). However, the mechanism is not completely known.

**Objective:** This study was aimed to explore the mitochondrial metabolism reprogrammed in AF patients by integrating metabolomics as well as proteomics of human atrium tissues.

**Methods and Results:** Left atrial tissue samples were harvested from 10 non-valvular AF patients and 10 matched samples from healthy donors for transplantation. In metabolomics analysis, 113 metabolites were upregulated and 10 metabolites were downregulated in AF, where multiple pathways related to mitochondrial energy metabolism were enriched. Correlation analysis between the differentially expressed proteins and metabolites identified several hub proteins related to mitochondrial function including Glycerol-3-phosphate dehydrogenase 2 (GPD2), Synemin (SYNM), Plectin (PLEC), with MCC score of 27, 17, 16, respectively, which have the most interactions with the dysregulated metabolites and ranked at the top in network string interactions scored by MCC method. All 330 differentially expressed proteins including 225 upregulated and 105 downregulated molecules were revealed and analyzed, which identified the downregulation of GPD2 (*p* = 0.02 and FC = 0.77), PLEC (*p* < 0.001 and FC = 0.71) and SYNM (*p* = 0.04 and FC = 0.76) in AF patients. Gene Set Variation Analysis (GSEA) showed mitochondrial metabolism-associated pathways including oxidative phosphorylation (NES: −1.73) and ATP biosynthetic process (NES: −2.29), were dramatically diversified in human AF. In GSVA, the expression levels of GPD2, PLEC, and SYNM were demonstrated to be associated with multiple metabolic pathways related to mitochondrial function (e.g., lipid metabolism and AMP activated protein kinase signaling) and cardiac structural and electrical remodeling (e.g., contractile fiber, ion homeostasis), which were proven vital in the development and maintenance of AF.

**Conclusion:** In all, this study provides new insights into understanding the mechanisms of AF progression, especially the reprogramming mitochondrial metabolism, and identifies several genes related to mitochondrial function as novel targets for AF, which may be involved in the occurrence and development of AF.

## Introduction

Atrial fibrillation (AF) is the most common arrhythmia managed in clinical practice with 22%–26% estimated lifetime risk, and is associated with substantial morbidity and increased mortality due to the combination of altered hemodynamics, atrioventricular dyssynchrony, progressive atrial and ventricular mechanical dysfunction, and thromboembolic complications (Hindricks et al., 2020; Brundel et al., 2022). The rising burden of AF, together with the significant increase in coexisting chronic cardiovascular conditions, is evidenced by a global uptrend in AF hospitalization (Kornej et al., 2020).

Atrial remodeling is considered as the most important mechanism involved in the development and progression of AF, including structural and electrical remodeling (Chen et al., 2021a). Recent studies show the metabolic changes of the atrium, especially the mitochondrial metabolism, significantly impact the electrical signaling and structural remodeling of the atrium, and play important roles in the occurrence and development of AF (Bai et al., 2019; Mason et al., 2020; Yuan et al., 2020). In addition, various metabolic disorders (i.e., obesity, diabetes mellitus, and hyperthyroidism) have been proved as risk factors of AF (Lavie et al., 2017; Wang et al., 2019; Lin et al., 2021), through the mechanism of mediating myocyte hypertrophy or fibrosis, *etc.* (Karam et al., 2017; Sagris et al., 2021). Thus, metabolic disorders play an important role in the occurrence and development of AF (Hajhosseiny et al., 2015; Zheng et al., 2021). However, it is unclear what kind of specific alternation of the metabolism profile is associated with the development of AF, indicating further explorations are required.

The omics technology can generate a large amount of multidimensional data that are amenable to be analyzed by new informatics methods, which have recently been applied to explore the molecular changes and identify the mechanism of AF-related remodeling (Jiang et al., 2019; Liu et al., 2020a; Roselli et al., 2020). As important supplements to genomics and transcriptomics, metabolomics and proteomics are more sensitive to external factors and can better reflect the real physiological state of biological systems (McGarrah et al., 2018; Liu et al., 2020b). In addition, the rapidly-developing mass spectrometry (MS)-based omics techniques provide new tools for numerous metabolite or protein level patterns in biological samples (Challen and Cramer, 2022) and offer more valuable data for biomarker screening and pathological exploration in AF, especially by multi-omics at the myocardial tissue level, for which data are limited.

In this study, integrated multi-omics analysis of metabolomics-proteomics of human atrium tissues were performed and analyzed to explore the metabolism reprogramed in AF patients and describe the key dysregulated metabolites and proteins as well as related metabolic pathways in AF patients relative to patients with sinus rhythm (SR).

## Methods

### Statement on human specimens

**FIGURE 1:**
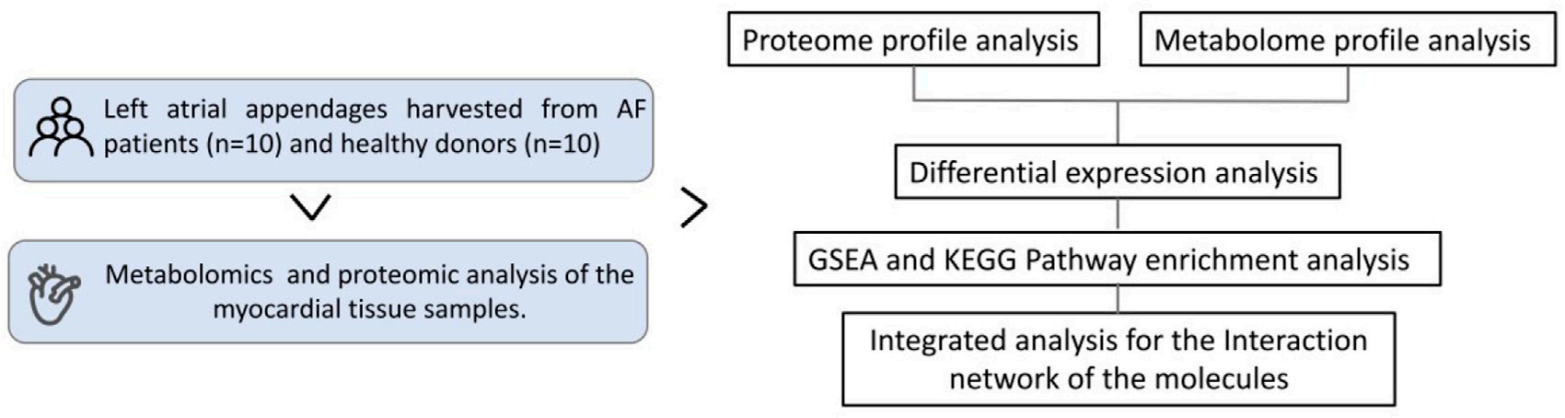
Study flowchart.

Left atrial appendages (LAAs) were harvested from AF patients undergoing surgical ablation of AF without cardiopulmonary bypass. Subjects (10 patients) diagnosed with non-valvular (without moderate to severe mitral valve stenosis or prosthetic valve) AF suffered from persistent AF for more than 1 year. For comparison, LAAs from 10 matched SR patients were collected. The controls in SR were matched by age and gender. Normal left atrial appendages were collected from healthy donors. The specimens obtained from the homograft bank had been rejected for clinic use and therefore allocated for research. As required in the research ethics, the clinical criteria for the healthy donors rejected for transplantation including failure in pairing between the donor and recipient of blood matching, *etc.*, or the recipient has been already evaluated not suitable for receiving transplantation anymore due to worsening condition with multiple organs failure and unable to tolerate the surgery, or the recipient had died before the donor arrived. The specimens were immediately snap-frozen and stored in liquid nitrogen (Figure 1). Related clinical information was collected from the Hospital Information System (HIS).

This study was approved by the Ethics Committee Board of Shanghai East Hospital (Approval No. 0402017), and performed in line with the Declaration of Helsinki.

### Proteomics and metabolomics analysis

Raw proteomics data were processed using MaxQuant_2.1.0.0, Perseus 2.0.7.0 and R package MSstats 4.4.1. Raw metabolomics data were analyzed using xcms 3.18.0. The overall proteomic and metabolic changes in AF were determined by OPLS-DA on R package mixOmics (Rohart et al., 2017). Additionally, univariate analysis of Wilcoxon Mann-Whitney U test was conducted for differential analysis.

Dysregulated metabolites and proteins were selected and mapped into the Small Molecule Pathway Database (SMPDB) (http://www.smpdb.ca/) and Kyoto Encyclopedia of Genes and Genomes (KEGG) database (http://home.jp/kegg/) for pathway enrichment analysis using R package MetaboAnalystR 3.2.0. Multi-omics analysis of pathways sharing both significant proteins and metabolites using MetaboAnalyst using up- or downregulated proteins and metabolites. Gene Set Enrichment Analysis (GSEA) was also used to analyze the correlations of different gene sets with GSEA 4.1.0. Gene Set Variation Analysis (GSVA) was used to study correlations using R package GSVA 1.44.5.

The correlative metabolites between metabolomics and proteomics were screened out by spearman correlation analysis using R package psych 2.2.9. All the nodes were loaded to CytoScape for network construction in the light of the correlation data.

### Statistical analysis

Data are presented as mean ± standard error of the mean (SEM) for at least 3 or 5 independent assays unless otherwise noted. All data passed the normality and equal variance before analysis. Student’s t-test was used for 2-sample comparison. One-way analysis variance (ANOVA) with Tukey *post hoc* tests was used for comparisons among multiple groups. Two-way ANOVA was used for comparisons among multiple groups when there were 2 experimental factors. *p* < 0.05 was considered as statistically significant. Bioinformatic analysis was performed using R 4.2.1 and Rstudio 2022.07.1 Build 554. Cytoscale 3.9.1 was used to construct networks for correlation analysis.

## Results

In this study, multiple targets and pathways related to mitochondrial function were unveiled and enriched by metabolomics and proteomics profiles of hearts from atrial fibrillation patients including oxidative phosphorylation and ATP biosynthetic process, *etc.*, which have vital functions in maintaining the homeostasis of the myocardium (Covian and Balaban, 2012; Mason et al., 2020), providing a comprehensive analysis of proteomics and metabolomics landscape of human AF.

**FIGURE 2:**
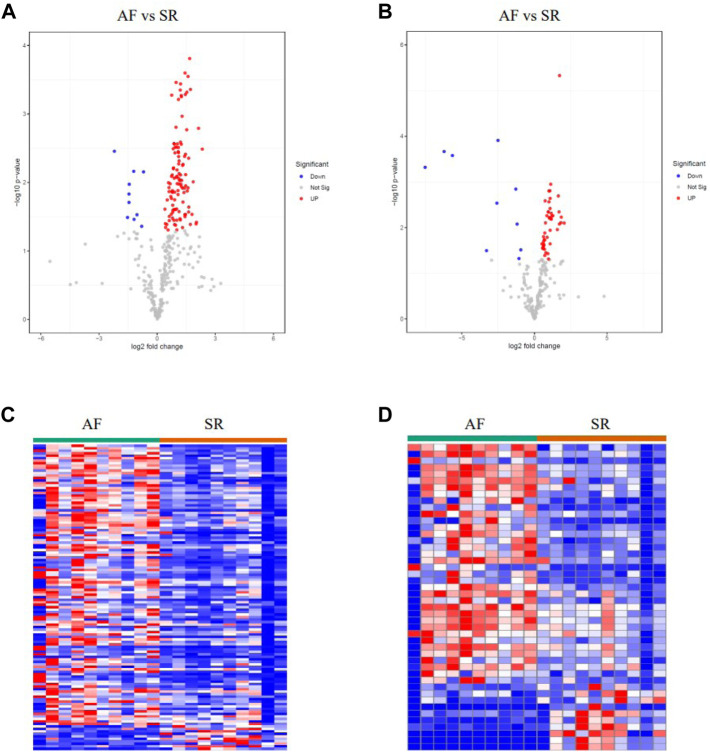
Metabolomic profiling analysis of AF samples and SR samples. **(A–B)** Volcano plots of the differently expressed metabolites in positively- and negatively-ionized modes, respectively. **(C–D)** Heatmaps of the differently expressed metabolites in positively- and negatively-ionized modes, respectively.

LAA samples were harvested from 10 persistent non-valvular AF patients during surgical ablation of atrial fibrillation and 10 SR samples matched by age and gender from healthy donors. Metabolomic analyses were then carried out using the 10 AF samples and the 10 SR samples. Differential metabolites were identified with the criteria of |logFC| > 0.263 and *p* < 0.05. There were 113 significantly upregulated metabolites and 10 significantly downregulated metabolites in AF patients compared to the SR group. Especially, the alterations of Raffinose, Adenine and D-Mannitol with FCs of 0.36, 0.44, and 0.22, respectively, are associated with mitochondrial energy metabolism (Figure 2 and Supplementary Table S1).

**FIGURE 3:**
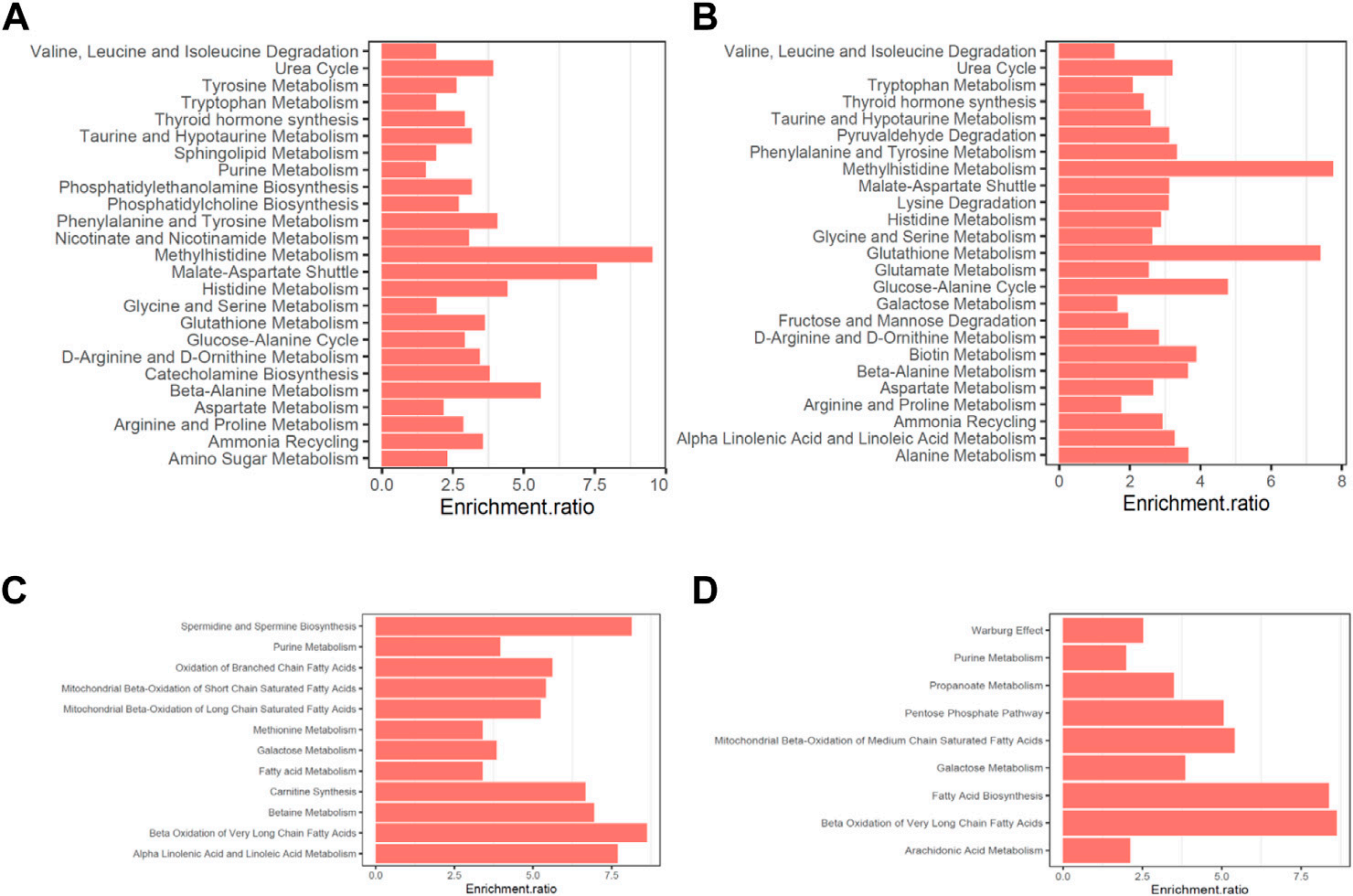
Pathway enrichments of differently expressed metabolites in non-valvular AF compared to SR group. **(A–B)** Pathways enrichened of the upregulated metabolites in the positively- and negatively-ionized modes, respectively. **(C–D)** Pathways enrichened of the downregulated metabolites in the positively- and negatively-ionized modes, respectively.

Moreover, the altered metabolites in AF were enriched in multiple pathways related to mitochondria and glycolipid metabolism. Notably, among the downregulated metabolites in both positively- and negatively-ionized modes in AF patients, pathways including mitochondrial beta-oxidation, fatty acid metabolism and galactose metabolism were enriched (Figure 3). Moreover, pathways associated with various important amino acid metabolisms were enriched, with the upregulated metabolites, such as beta-alanine metabolism, histidine metabolism, and glycine and serine metabolism (enrichment scores 5.58, 4.425 and 1.923 respectively).

**FIGURE 4:**
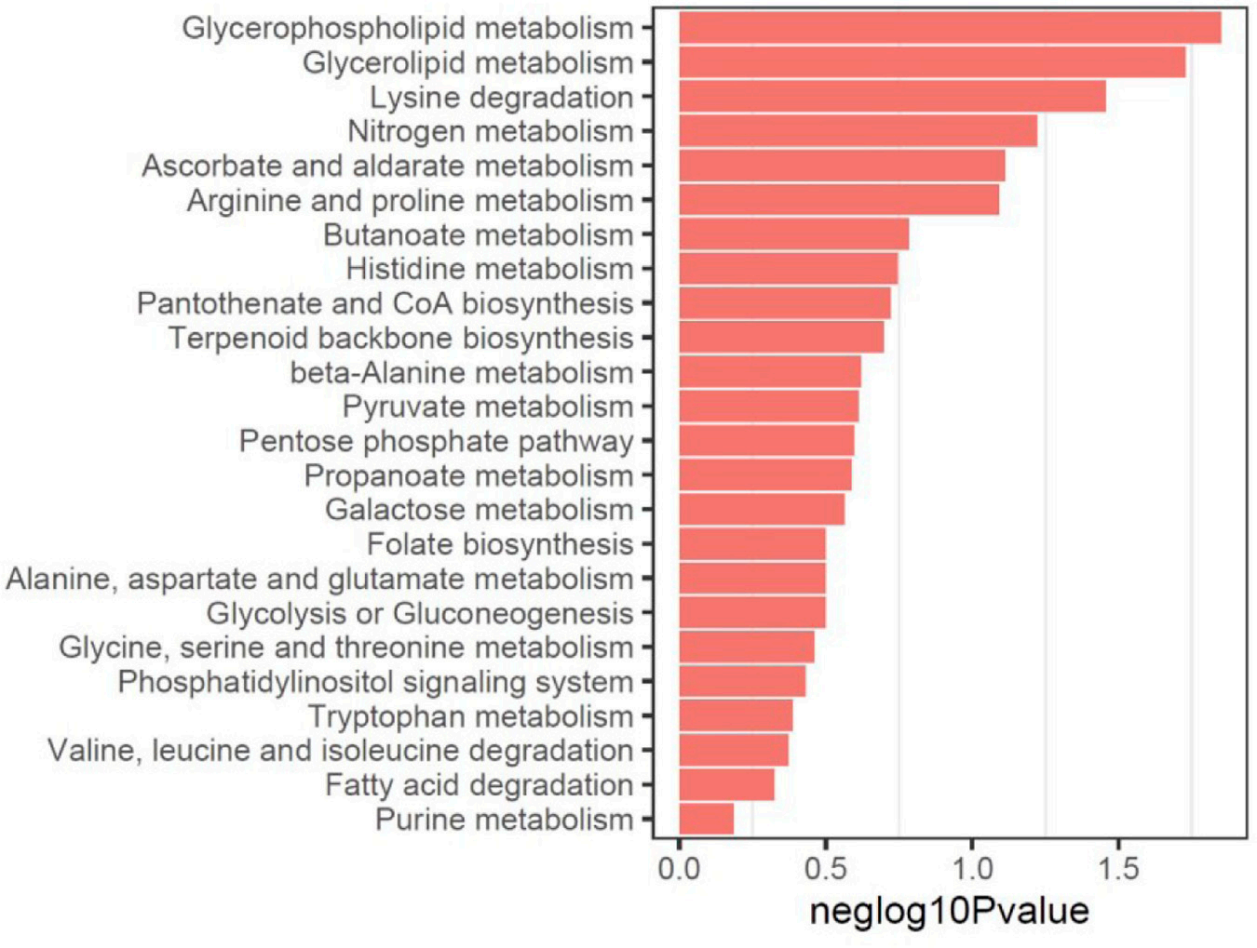
Multi-omics analysis of pathways sharing both significant proteins and metabolites using MetaboAnalyst using downregulated proteins and metabolites.

Analysis of pathways sharing both significant proteins and metabolites using MetaboAnalyst was then performed. As a result, multiple glycolipid metabolic pathways, such as glycerophospholipid metabolism and glycerolipid metabolism were mostly enriched using the downregulated proteins and metabolites (Figure 4), and various important amino acid metabolic pathways were significantly enriched using the upregulated metabolomics and proteomics profiles (Supplementary Figure S1), consistent with the results of metabolites analysis.

**FIGURE 5:**
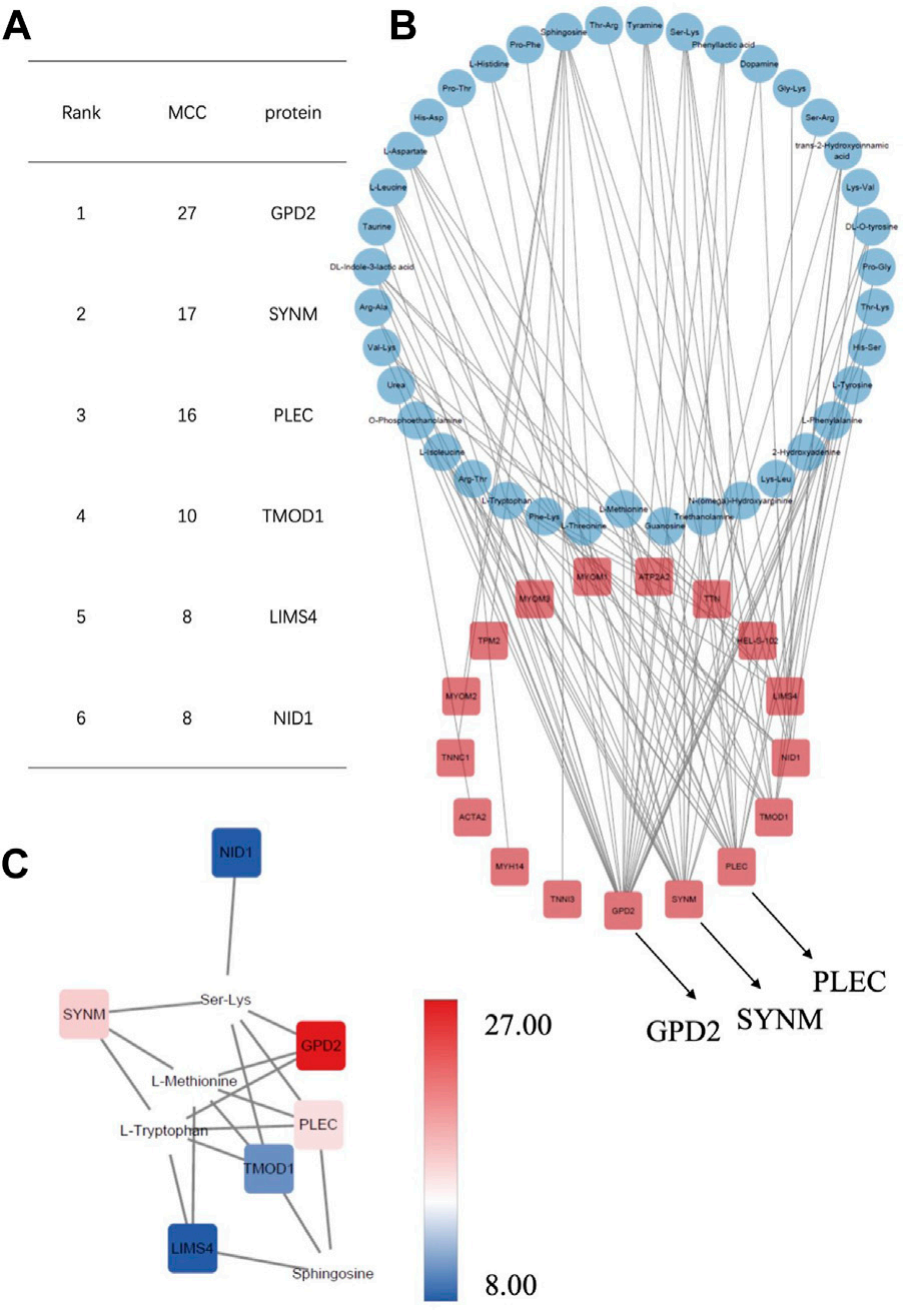
Correlation analysis of the differentially expressed proteins with metabolites in positively-ionized mode.

Proteomics samples and metabolomic samples were then sent to correlation analysis using interaction networks of proteins with *p* < 0.05 and metabolites with VIP >1 and *p* < 0.05. Correlations with the top 100 r values and *p* < 0.05 were next constructed, which demonstrated obvious clustering patterns of proteins and metabolites that represented the metabolism changes in AF. Network diagram was drawn on Cytoscape by sorting the absolute values of correlation coefficient (Figure 5B, Supplementary Figure S2 and Supplementary Tables S2, S3). As a result, several hub proteins with metabolites were identified, among which glycerol-3-phosphate dehydrogenase 2 (GPD2) was found at the top of the network string interactions ranked by maximal clique centrality (MCC) method in positively-ionized mode (MCC score = 27; Figures 5A, C), which is located at the inner membrane of mitochondria and closely related to mitochondria energy metabolism (Mráček et al., 2013; Ishihama et al., 2021; Liu et al., 2021). Together with GPD2, SYNM ranked at the top three (MCC score = 17,16, respectively) as well, which have also been reported to be associated with mitochondrial function (Winter et al., 2015; Kuznetsov et al., 2020; Paulin et al., 2020; Baek et al., 2021), indicating its potential participation of mitochondria metabolism in AF.

**FIGURE 6:**
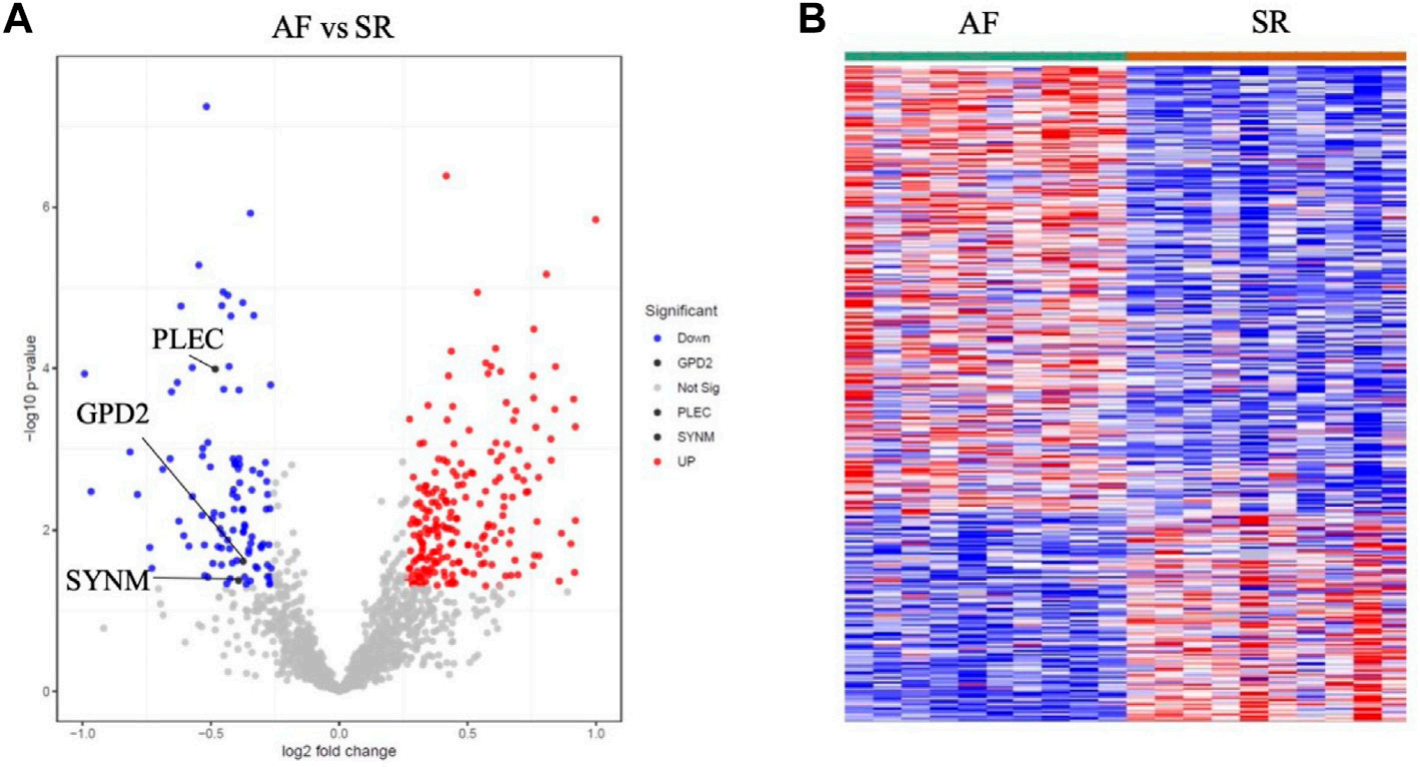
Proteomics profiling analysis of AF and SR samples showing the downregulation of GPD2, PLEC and SYNM in the volcano plot **(A)** and heatmap **(B)**.

Proteomics samples were tested by univariate analysis using the criteria for differentially expressed proteins (DEPs): *p* < 0.05 and logFC >0.263 for upregulation, and *p* < 0.05 and logFC < −0.263 for downregulation. All 330 DEPs including 225 upregulated DEPs and 105 downregulated DEPs were analyzed, which identified the downregulation of GPD2 (*p* = 0.02 and FC = 0.77), PLEC (*p* < 0.001 and FC = 0.71) and SYNM (*p* = 0.04 and FC = 0.76) in AF patients in AF patients (Figure 6 and Supplementary Table S4).

**FIGURE 7:**
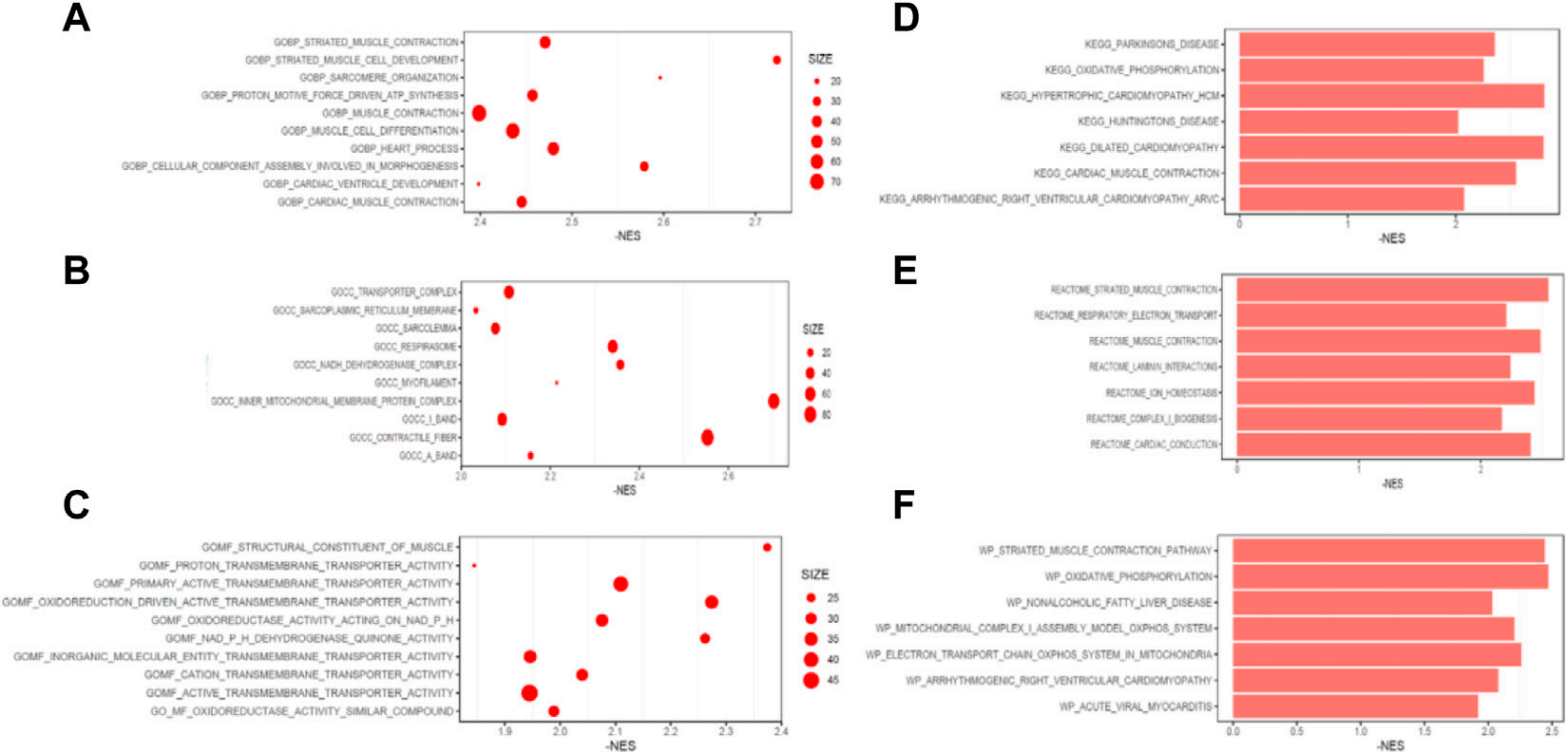
Pathways enriched of the differentially expressed proteins. Top 10 pathways enriched by GO for biological processes **(A)**, cellular components **(B)** and molecular function **(C)**. Top 7 pathways enriched by KEGG **(D)**, REATOME **(E)** and WIKI **(F)**.

Pathway enrichment analysis was then performed. For biological processes (BP) by GO analysis, DEPs were enriched in heart development, respiratory electron transport chain and actomyosin structure organization (Figures 7A–C). For cellular components (CC), DEPs were enriched in mitochondrial protein containing complex, organelle inner membrane, and mitochondrial proton transporting ATP synthase complex coupling factor. For molecular functions (MF), DEPs were enriched in proton transmembrane transporter activity, active transmembrane transporter activity and inorganic molecular entity transmembrane transporter activity.

**FIGURE 8:**
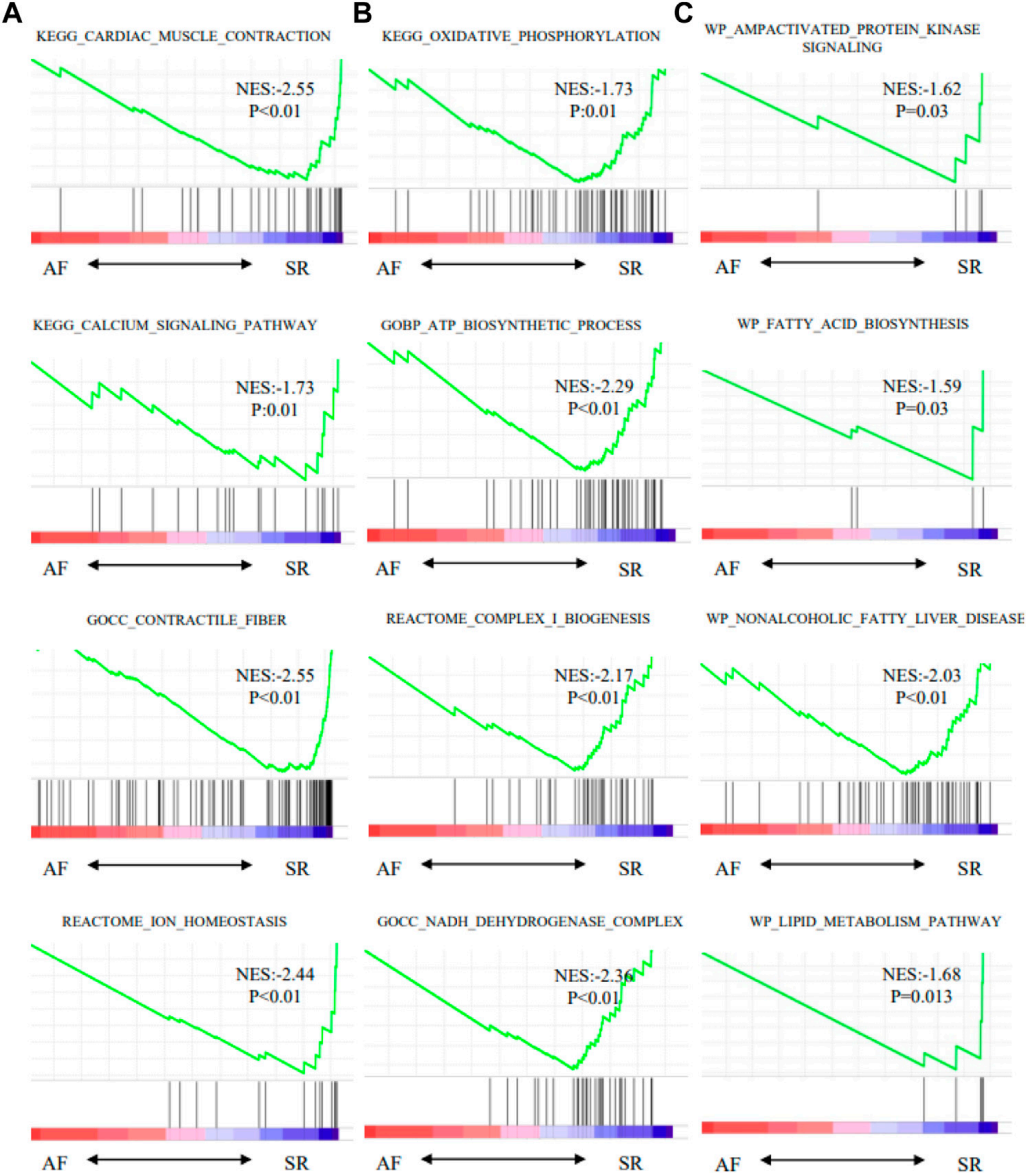
Pathways enriched by GSEA. **(A)** Characteristics of AF. **(B)** Mitochondrial metabolism pathway. **(C)** Glycose and lipid metabolism pathway.

Moreover, oxidative phosphorylation and cardiac muscle contraction were enriched by KEGG (Figure 7D). Ion channel transport, and organelle biogenesis and maintenance were enriched by REACTOME (Figure 7E). Oxidative phosphorylation was enriched with the largest score (NES = −2.47) by WIKI (Figure 7F). GSEA for function enrichment showed several pathways related to mitochondrial energy metabolisms were significantly downregulated in AF samples, including oxidative phosphorylation (*p* < 0.01), ATP biosynthetic process (*p* < 0.01) and NADH dehydrogenase complex (*p* < 0.01) (Figure 8), which were also reportedly associated with the function of GPD2 (Mráček et al., 2013; Ishihama et al., 2021; Liu et al., 2021).

**FIGURE 9:**
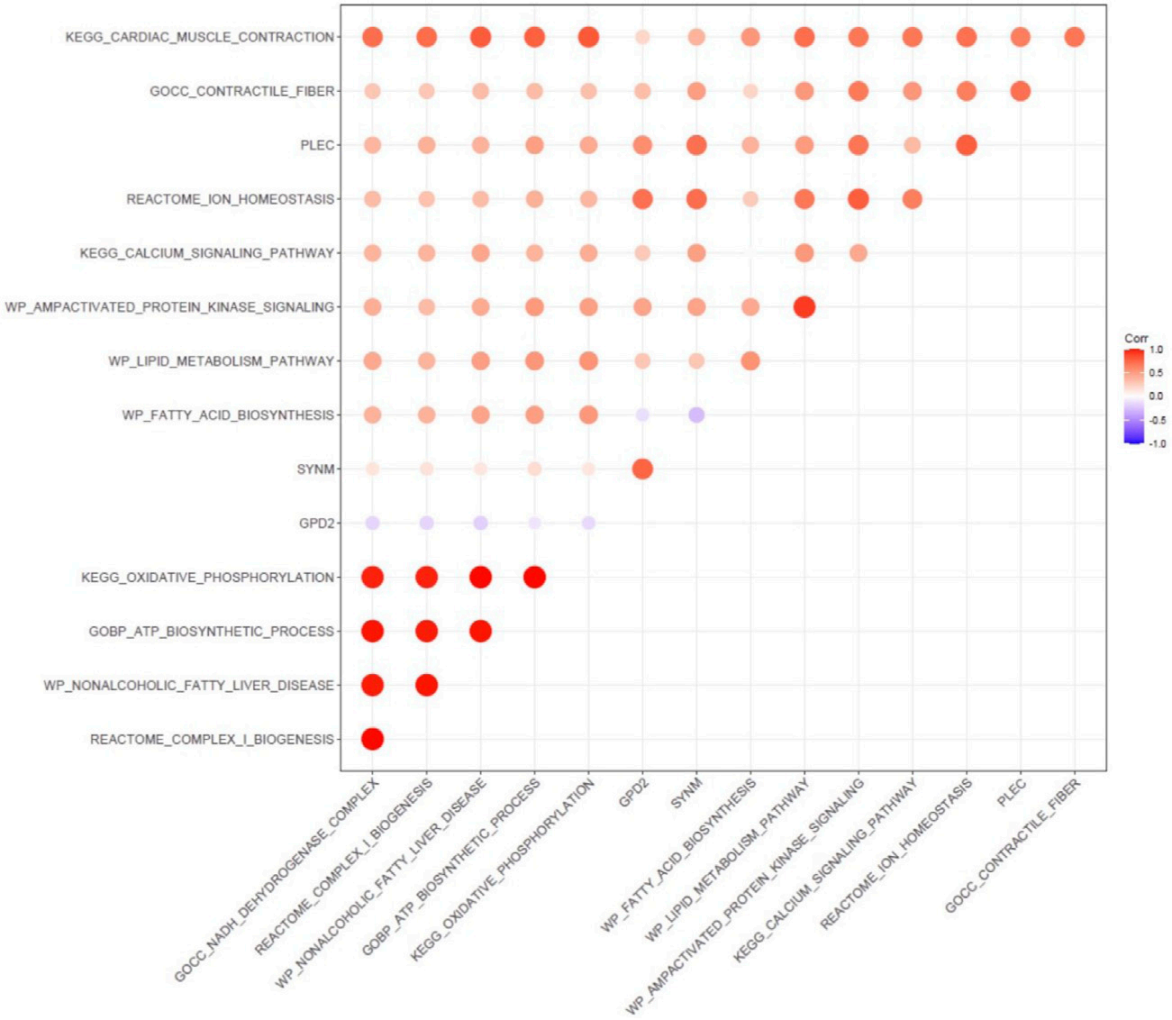
Gene set variation analysis (GSVA) for correlation of enriched gene sets.

Gene Set Variation Analysis (GSVA) was then carried out to analyze the correlation of enriched gene sets and the expression of GPD2, PLEC, and SYMN (Figure 9). The GPD2 expression level was associated with multiple pathways related to cardiac structural remodeling [e.g., contractile fiber (*r* = 0.33), cardiac muscle contraction (*r* = 0.2)], and various pathways relevant to myocardial electrical remodeling, including calcium signaling (*r* = 0.27) and ion homeostasis (*r* = 0.7), and metabolic pathways such as lipid metabolism (*r* = 0.28) and AMP activated protein kinase signaling (*r* = 0.46). The expression levels of SYNM showed association with pathways related to cardiac remodeling and energy metabolism [e.g., cardiac muscle contraction (*r* = 0.38), ion homeostasis (*r* = 0.71), lipid metabolism (*r* = 0.28)], and PLEC demonstrated association with pathways related to mitochondria function [e.g., oxidative phosphorylation (*r* = 0.43), ATP biosynthetic process (*r* = 0.48)].

## Discussion

### Metabolism reprogramming in AF

AF is a source of significant morbidity and mortality associated with heart failure, stroke, and other complications (Hindricks et al., 2020; Brundel et al., 2022). Atrial remodeling including structural and electrical remodeling is among the most important mechanisms involved in the development and progression of AF (Chen et al., 2021a).

AF-induced irregular heartbeat alters atrial hemodynamics, oxygen delivery, and energy supply, which all influence the metabolic state (Kolwicz et al., 2013; Chistiakov et al., 2018). Recent studies demonstrate substantial changes in energy metabolism and metabolism-related enzymes in AF patients compared to patients in sinus rhythm (SR) (Bai et al., 2019; Mason et al., 2020; Yuan et al., 2020). Strong evidence shows that these maladaptive metabolic remodeling activities may represent a driving force for atrial electrical or structural remodeling (McCauley et al., 2020; Chen et al., 2021b; Fang et al., 2022). In a cross-sectional observational study, AF patients had more metabolic complications than the control population matched for age and sex, which are supposed to induce the AF substrate or as a result of AF (Boos, 2020). Nevertheless, the detailed mechanisms remain unclear.

### Advantages of multiomics analysis using proteomics and metabolomics

Metabolomics, an emerging type of omics, reflects the downstream products of gene expression and enables a more direct and accurate reflection of the pathophysiological state of an organism. Metabolomics is widely used in new drug development, disease diagnosis, and personalized therapy (Johnson et al., 2016). Proteomics is widely applied into research at large-scale protein levels, and enables researchers to investigate the protein alterations that lead to the pathological progression of diseases (Lindsey et al., 2015). The combination of proteomics and metabolomics enables the analysis of proteins and metabolites involved in the same metabolic pathway, and better reveals the biological processes that an organism produces after a disease or external stimulus, so as to comprehensively and insightfully understand disease processes, cellular physio-pathological processes, and regulatory networks to reveal the basic rules of life activities.

### Metabolomics and proteomics of AF heart tissue

Recent studies show mitochondrial metabolism, the most predominant functional site of energy metabolism in cardiomyocytes, plays an important role in the development of AF (Brown et al., 2021; Yuan et al., 2022). It also regulates the oxidative phosphorylation of various metabolites and influences the contraction and ion homeostasis of myocardial tissues (Avula et al., 2021). Nevertheless, further exploration is still needed.

In this study, multiple metabolites are significantly dysregulated in AF, such as the alterations of Raffinose, Adenine and D-Mannitol with FCs of 0.36, 0.44, and 0.22, respectively, and are associated with mitochondrial energy metabolism. Moreover, the compensatory relations between amino acid metabolism and glucose-fatty metabolism have been observed in various diseases, which is very important for the energy metabolism homeostasis (Zhou and Tian, 2018; Careau et al., 2021). Recent studies have revealed the disorder of glycolipid metabolism in AF cardiac tissue, such as the Warburg effect, *etc.* (Hu et al., 2019; Liu et al., 2019). In this study, among the downregulated metabolites in AF, pathways related to mitochondrial beta-oxidation, fatty acid metabolism and galactose metabolism were enriched. On the contrary, using the upregulated metabolites, pathways associated with various important amino acid metabolisms were enriched, which may serve as compensatory mechanism for the energy metabolism.

In the proteomics analysis, mitochondria-associated pathways were also significantly enriched using multiple methods, such as the oxidative phosphorylation, ATP biosynthetic process and NADH dehydrogenase complex, indicating the mitochondrial dysfunction may play an important role in the development and maintenance of AF.

### Multiple targets related to mitochondrial function uncovered by multi-omics

In the correlation analysis between the differentially expressed proteins and metabolites, several hub proteins related to mitochondrial function were identified, including GPD2, PLEC, and SYNM, which has the most interactions with the dysregulated metabolites and ranked at the top in network string interactions scored by MCC method.

In this study, GPD2 demonstrated with the most interactions with the dysregulated metabolites in both positively- and negatively-ionized modes, which is known as an integral component of the respiratory chain located at inner mitochondrial membrane and connecting mitochondrial and cytosolic processes *via* the glycerophosphate (GP)-shuttle, playing an important role in cell energy metabolism (Ishihama et al., 2021; Liu et al., 2021). In addition, it participates in multiple cardiac diseases *via* regulating mitochondrial function (Mráček et al., 2013). PLEC is a versatile cytolinker protein that has been found to be intimately associated with mitochondria and may participate in several cardiac diseases (Winter et al., 2015; Kuznetsov et al., 2020; Baek et al., 2021). SYNM is a new candidate gene and shows numerous sequence polymorphisms, which is considered as an intermediate filament-associated protein and has interconnects with cardiac mitochondria (Paulin et al., 2020). However, their roles in studies on AF remain unclear.

In GSVA, the expression levels of GPD2, PLEC, and SYNM demonstrated association with multiple metabolic pathways related to mitochondrial function (e.g., lipid metabolism and AMP activated protein kinase signaling) and cardiac structural and electrical remodeling (e.g., contractile fiber, ion homeostasis), which were proven vital for the development and maintenance of AF (Hindricks et al., 2020; Brundel et al., 2022). Moreover, GSVA demonstrated the metabolism reprogramming was closely related to atrial electrical and structural remodeling, indicating its potential role in AF.

In addition, the limitations of this study include that analysis performed with different tools or metrics has not been compared and discussed in this study and function validation based on experimental approach should be carried out in the further study.

## Conclusion

This study provides new insights into the mechanisms of AF progression, especially the reprogramming mitochondrial metabolism, and identifies multiple genes related to mitochondrial function as novel targets for non-valvular AF, which may participate in and maintain AF.

## Data Availability

The data presented in the study are deposited in the ProteomeXchange Consortium (http://proteomecentral.proteomexchange.org) *via* the iProX partner repository, accession number PXD041147.

## Abbreviations

AF, Atrial Fibrillation; BSA, Body surface area; CAD, Coronary artery disease; DEGs, Differential expression genes; DEPs, Differentially expressed proteins; DM, Diabetes mellitus; ECG, Electrocardiograph; GPD2, Glycerol-3-phosphate dehydrogenase 2; GSVA, Gene Set Variation Analysis; HIS, Hospital Information System; HTN, Hypertension; KEGG, Encyclopedia of Genes and Genomes; LAA, Left atrial appendages; LAD, Left atrial diameter; LVEF, Left ventricular ejection fraction; MS, Mitral stenosis; MV, Mitral valve; NAFLD, Non-alcoholic fatty liver disease; NYHA, New York Heart Association; PLEC, Plectin; SR, Sinus Rhythm; SYNM, Synemin.
