# Supplementary material for: Multiple targets related to mitochondrial function unveiled by metabolomics and proteomics profiles of hearts from atrial fibrillation patients
Source: Front Physiol. 2023 Apr 4;14:1123391. doi: 10.3389/fphys.2023.1123391 (PMC10110950; doi:10.3389/fphys.2023.1123391)
Supplement: Supplementary file 3 [file Table1.DOCX]

Supplementary Material

**Multiple targets related to mitochondrial function unveiled by metabolomics and proteomics profiles of hearts from atrial fibrillation patients**

**Key Words:** Atrial fibrillation, Mitochondrial function, Multiple targets, Proteomics, Metabolomics.

The PDF file includes:

1. Supplementary Table S1 S4
2. Supplementary Figure S1-2
3. **Supplementary Table S1 S4**

**Supplementary Table S1**：

Dysregulated metabolites included in the heatmaps presented in Figure 2 (C-D).

|  | Dysregulated metabolites |
| --- | --- |
| Positively-ionized mode (123) | Warfarin, Trimethylamine N-oxide, Ergothioneine, Tyr-Phe, His-Thr, D-Alanyl-D-alanine (D-Ala-D-Ala), Phe-Lys, Phe-Arg, Arg-Met, L-Tryptophan, Pro-Arg, DL-Indole-3-lactic acid, beta.-Homoproline, L-Histidine, Arg-Val, Pro-Gly, N-Acetyl-L-phenylalanine, trans-2-Hydroxycinnamic acid, MG(18:2(9Z,12Z)/0:0/0:0)[rac], DL-O-tyrosine, Phe-Gln, Ser-Phe, L-Aspartate, Phe-Trp, Quinone, Triethanolamine, L-Tyrosine, 4-Hydroxycinnamic acid, Ile-Lys, Dopamine, Pro-Phe, Ile-Phe, Ile-Ala, Ile-Val, Lys-Lys, His-Ile, L-Glutamate, His-Ser, Val-His, Lys-Leu, Val-Lys, Creatine, Phe-Ala, Arg-Lys, Ile-Asn, Thr-Arg, Thr-His, Val-Tyr, Lys-Val, 1-Methylhistidine, 2-Hydroxyadenine, Phe-Gly, N-Acetylneuraminic acid, Guanosine, L-Phenylalanine, Tyramine, Gly-Thr, L-Threonine, O-Phosphoethanolamine, Arg-Ala, DL-Methionine sulfoxide, L-Methionine, Phenol, Pro-His, Phenyllactic acid, N-Acetylaspartylglutamate (NAAG), N-(omega)-Hydroxyarginine, Leu-Ala, His-Ala, Pro-Asn, Urea, Ser-Lys, 1-Oleoyl-sn-glycero-3-phosphocholine, Ala-Phe, L-Leucine, Ile-Ser, D-Erythrose 4-phosphate, His-Asp, Thr-Lys, Leu-Glu, Adenosine 3'-monophosphate, Lys-Ser, Taurine, Lys-Thr, Ser-Arg, Ser-Asp, L-Serine, L-Asparagine, Arg-Thr, gamma-L-Glutamyl-L-phenylalanine, Val-Asn, Ser-Ala, His-Pro, Pro-Thr, L-Carnosine, Pro-Trp, Sphingosine, L-Isoleucine, His-Gly, Pyridoxal (Vitamin B6), L-Alanine, gamma-L-Glutamyl-L-glutamic acid, sn-Glycerol 3-phosphoethanolamine, Thr-Thr, L-Pyroglutamic acid, Flavin adenine dinucleotide (FAD), L-Lysine, Histamine, Nicotinamide, Gly-Lys, (-)-Riboflavi, Nitrobenzene, 2(1H)-Pyridinone, 4-Aminobutyric acid, Diethanolamin, Pro-Met, Adenine, 1-Aminocyclopropanecarboxylic acid, (+-)8,9-DHET, Raffinose, 16-Hydroxypalmitic acid, Gly-Gln, D-Mannitol. |
| Negatively-ionized mode (56) | (+)-Warfarin, Indole, Alpha-N-Phenylacetyl-L-glutamine, L-Carnosine, Cytidine 2',3'-cyclic phosphate, Indoxyl sulfate, D-Alanyl-D-alanine (D-Ala-D-Ala), L-Tryptophan, L-Alanine, L-Tyrosine, Guanosine, Dihydrothymine, GDP-L-Fucose, L-Leucine, Uridine 5'-monophosphate (UMP), N-Acetylaspartylglutamate (NAAG), N-Acetyl-DL-methionine, DL-Methionine sulfoxide, L-Phenylalanine, D-Aspartic acid, Pantothenate, 3'-O-methylguanosine, L-Valine, Indole-3-carboxylic acid, Pseudouridine, L-Methionine, Glutathione disulfide, Glycerol, Ribothymidine, Adenosine 3'-monophosphat, D-Proline, 2-Oxoadipic acid, Oleic acid, D-Lyxose, Phosphorylcholine, L-Threonine, O-Phosphoethanolamine, sn-Glycerol 3-phosphoethanolamine, L-Pyroglutamic acid, L-Arginine, (S)-2-aminobutyric acid, L-Lysine, L-Glutamate, DL-Serine, Glycerol 3-phosphate, Lumichrome, 9R,10S-EpOME, 6-Phospho-D-gluconate, 9,10-DiHOME, DL-lactate, Diglykokoll, 2-hydroxy-butanoic acid, L-Iditol, D-Threitol, L-Rhamnose, Thiopental. |

**Supplementary Table S4**：

Differential expression proteins included in the heatmaps presented in Figure 6 B.

| DEPs | Gene Symbols |
| --- | --- |
| Upregulated (225) | APOE, LTBP2, DKK3, NTN1, CFHR1, CSPG2, HEL-S-92n, VTN, LYZ, C1QC, GPX1, TIMP3, FN1, EHD3, APOB, ABI3BP, ITIH2, MYH10, HSPA2, FGA, C8A, TNXB, MYOC, C8G, STING1, CLU, PPP1R12C, HMGN3, HEL-S-78p, OXCT1, PDXK, TGFBI, JCHAIN, FTH1, AFM, GPD1L, CTSZ, HEL-S-30, CALD1, PDLIM7, CMPK1, A2M, hCG_40889, NES, AKAP12, FTL, TMEM43, C9, PICALM, HEL-S-37, FLNA, MTPN, RBP4, PON1, ARHGDIB, CTTN, NAP1L1, COL6A1, S100A4, APOA4, PTMA, HEL-S-21, SGTA, TSFM, C5, VWF, ARHGAP1, YWHAQ, NME2, C4A, TXNDC5, SERPING1, STK38L, SERPINC1, HEL-S-304, SPR, RRBP1, NHLRC2, SLC16A7, SERPINB9, CPPED1, DPYSL3, MSRB3, ANXA4, CD109, PRKG1, HEL-S-165mP, UBE1, TXNRD2, DNAJA4, COL6A3, GSTM3, CSDA, NANS, GNG7, ITIH4, HEL57, MYL6, RNPEP, HEL11, PSMD13, TBRG4, LRP1, SARS, LAMTOR2, PTBP1, VWA1, ZADH2, HEL-S-2, HEL-S-106, DUSP, GPX4, COPG1, UBE1C, ACTN4, HEL-S-62p, AK4, DDAH1, TOM1, SEPTIN11, SDC2, LDHB, MSRB2, SEPTIN2, EIF4H, HSPA4, PDLIM3, HPRT1, APEX1, ALDH9A1, CAP1, YWHAZ, ARPC2, ALDH6A1, CAST, LDHD, IMPA1, PMPCA, IDH3A, GNG12, HEL-S-43, FXN, TXNRD1, PSMD12, EPHX2, hCG_1991735, ZYX, ATPAF2, ADRM1, HEL20, GLOD4, HEL-75, MAP4, DPYSL2, ARF1, PAICS, COPB, ANXA1, ATP6V1A, EIF2S1, PGAM1, ACTN1, GCDH, CCDC141, PPA2, FKBP1A, RPL24, RANBP5, PPP5C, ENO2, ACTR2, CRYZ, ISCU, SUCLG2, NUTF2, RAC1, RAP1B, PRMT1, PC4, VDP, ACADSB, SUCLG1, PSMA4, XRCC5, HEL-S-32, HEL-S-277, APPL1, ACO2, PKM2, VAMP3, CLIC1, HEL-S-71, CUTA, PSMD3, TPM4, ARHGDI, HEL-S-67p, NFS1, ACTR3, MUT, COPS7A, RAB11B, NSFL1C, PTPN11, HEL-S-68p, AGRN, HNRPD, TPP2, ETFB, IDH3B, PAK2, TGFB1I1, HMGB1, NAP1L4, HNRNPU, HNRPA3, C1QBP, ACADS, FLII, RAB1A, H2AFY, PSMC, TLN1, CYFIP1, CORO1B |
| Downregulated (105) | RAB10, RYR2, HEL-S-107, NEBL, ATP5PD, SLMAP, UQCRC1, CANX, PANK4, PCBD2, NDUFB6, ATP5J, TLN2, SRI, PRKAA2, MYH14, OBSCN, GUP1, ERAP1, JPH2, NID2, PHKA1, C1orf24, ATP5F1EP2, ACTA2, NDUFS4, SUI1, TRIM54, CDH13, BCAP29, HSPB7, LIMS4, ERLIN2, PRKACA, GPD2, TMEM205, TTN, SGCG, hCG_40688, SNTA1, DYSF, SNX1, SYNM, NID1, MYBPC3, DDT1, ATP2A2, LAMC1, ACTN2, HEL-S-102, HSPA12B, MYOZ2, HRC, PTGFR, TMOD1, MPC1, DHRS7, PFDN6, MYH6, ATP5F1D, ALDH3A2, SMYD1, MYOM1, C18orf55, MYOM3, UQCRB, ABLIM1, GNG, CHDH, DGK, PLEC, ILVBL, SGCE, FXR1, TNNC1, LDB3, CYTB, TNNI3, hCG_1773630, FLNC, PCYOX1, TPM2, TMEM143, MYOM2, DKFZp686P18250, LAMA2, UNQ5809, ATP6, LAMB1, SRL, CUL1, TNNT2, SCCPDH, CASQ2, MYH2, CA4, HEL-S-134P, LGALS3BP, TRDN, MYBPHL, COL28A1, PLIN1, MYL5, GPD1, MYL2. |

1. **Supplementary Figure S1**

**
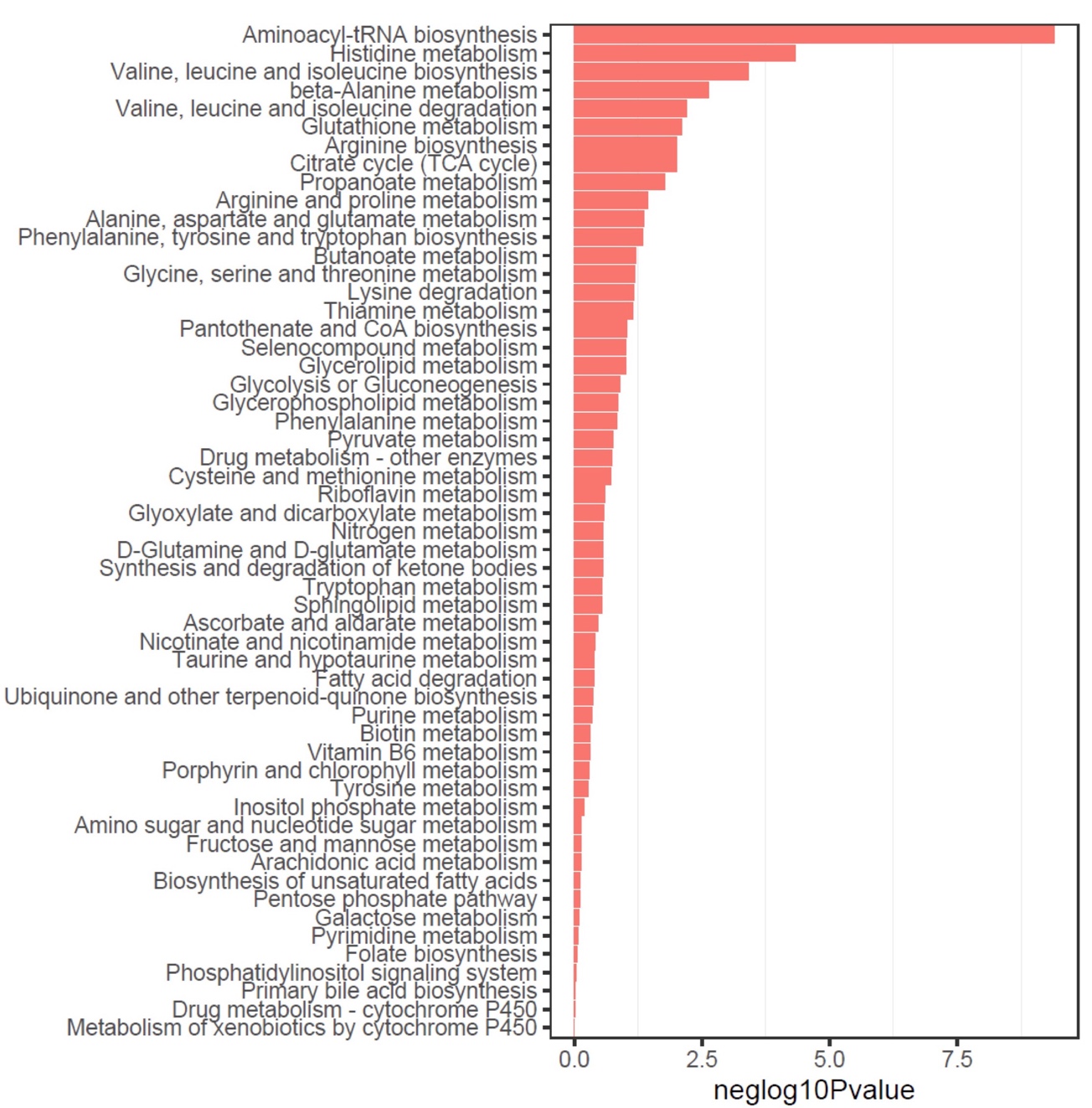
**

Figure S1: Multi-omics analysis of pathways sharing both significant proteins and metabolites using MetaboAnalyst using upregulated proteins and metabolites.

**Supplementary Figure S2**


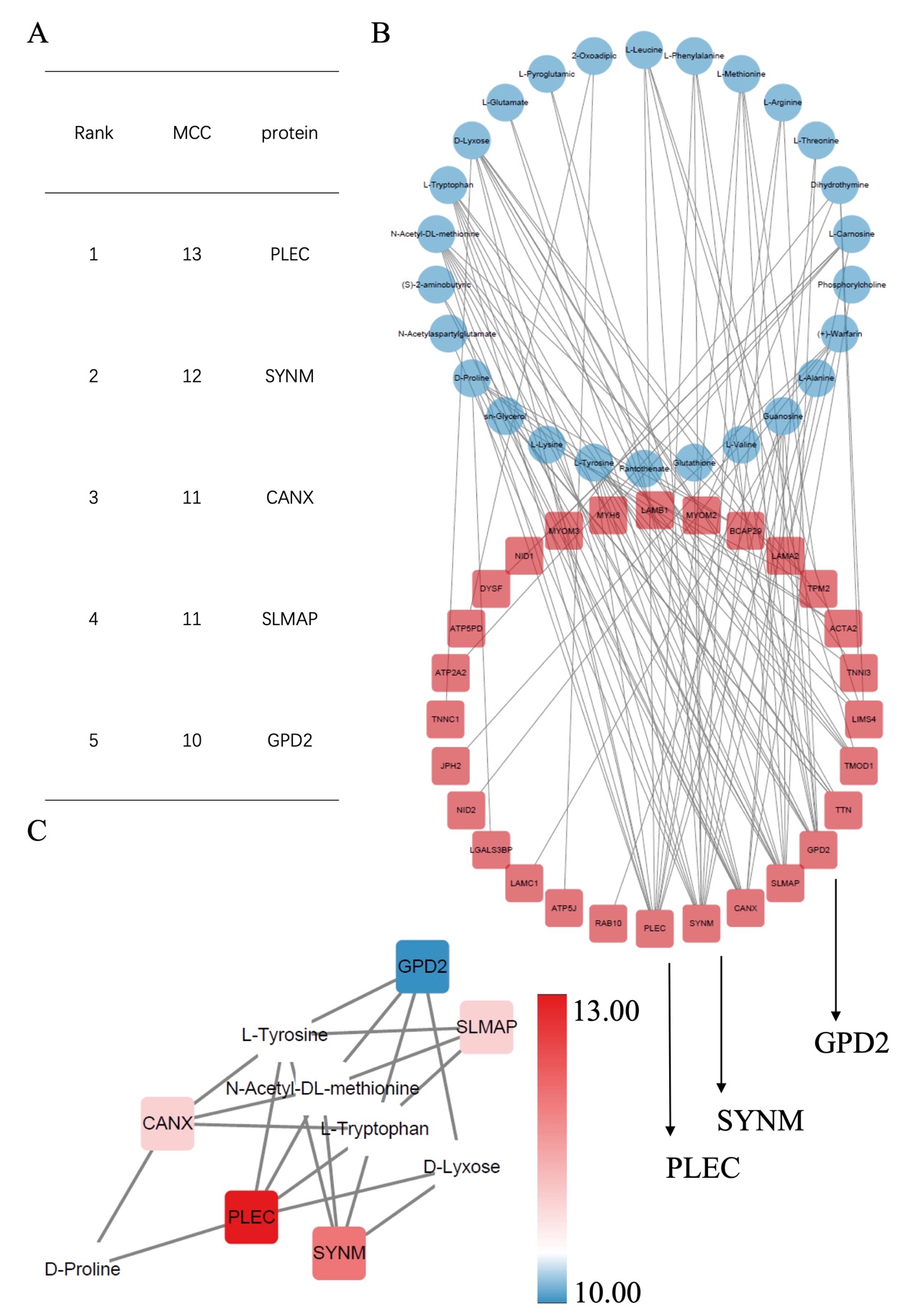


Figure S2: Correlation analysis of the differentially expressed proteins with metabolites in negatively-ionized modes.
